# Premorbid β1-selective (but not non-selective) β-blocker exposure reduces intensive care unit mortality among septic patients

**DOI:** 10.1186/s40560-021-00553-9

**Published:** 2021-05-13

**Authors:** Ming-Jen Kuo, Ruey-Hsing Chou, Ya-Wen Lu, Jiun-Yu Guo, Yi-Lin Tsai, Cheng-Hsueh Wu, Po-Hsun Huang, Shing-Jong Lin

**Affiliations:** 1 Division of Cardiology, Department of Medicine, Taipei Veterans General Hospital, Taipei, Taiwan; 2 Cardiovascular Research Center, National Yang Ming Chiao Tung University, Taipei, Taiwan; 3 Department of Critical Care Medicine, Taipei Veterans General Hospital, 112, No. 201, Sec. 2, Shih-Pai Road, Taipei, Taiwan; 4 Institute of Clinical Medicine, National Yang Ming Chiao Tung University, Taipei, Taiwan; 5 Department of Medical Research, Taipei Veterans General Hospital, Taipei, Taiwan; 6 Taipei Heart Institute, Taipei Medical University, Taipei, Taiwan; 7 Division of Cardiology, Heart Center, Cheng-Hsin General Hospital, Taipei, Taiwan

**Keywords:** Sepsis, *β-blocker*, β1-selective *β-blocker*, *Tachycardia*, *Catecholamine*, *Intensive care unit*

## Abstract

**Background:**

*β-blocker*s may protect against catecholaminergic myocardial injury in critically ill patients. Long-term *β-blocker* users are known to have lower lactate concentrations and favorable sepsis outcomes. However, the effects of β1-selective and nonselective *β-blockers* on sepsis outcomes have not been compared. This study was conducted to investigate the impacts of different *β-blocker* classes on the mortality rate in septic patients.

**Methods:**

We retrospectively screened 2678 patients admitted to the medical or surgical intensive care unit (ICU) between December 2015 and July 2017. Data from patients who met the Sepsis-3 criteria at ICU admission were included in the analysis. Premorbid *β-blocker* exposure was defined as the prescription of any *β-blocker* for at least 1 month. Bisoprolol, metoprolol, and atenolol were classified as β1-selective *β-blockers*, and others were classified as nonselective *β-blockers*. All patients were followed for 28 days or until death.

**Results:**

Among 1262 septic patients, 209 (16.6%) patients were long-term *β-blocker* users. Patients with premorbid *β-blocker* exposure had lower heart rates, initial lactate concentrations, and ICU mortality. After adjustment for disease severity, comorbidities, blood pressure, heart rate, and laboratory data, reduced ICU mortality was associated with premorbid β1-selective [adjusted hazard ratio, 0.40; 95% confidence interval (CI), 0.18–0.92; *P* = 0.030], but not non-selective *β-blocker* use.

**Conclusion:**

Premorbid β1-selective, but not non-selective, *β-blocker* use was associated with improved mortality in septic patients. This finding supports the protective effect of β1-selective *β-blockers* in septic patients. Prospective studies are needed to confirm it.

**Supplementary Information:**

The online version contains supplementary material available at 10.1186/s40560-021-00553-9.

## Introduction

Sepsis, defined as organ dysfunction caused by a dysregulated host response to infection [1], is a leading cause of death in the intensive care unit (ICU). Despite significant advances in intensive care medicine, septic shock mortality rates remain high, ranging from 40 to 50% [1]. Hence, more knowledge of the pathophysiology of sepsis is needed. Overwhelming inflammation, arterial vasodilation, and hypovolemia are the main components of the early phase of sepsis. Sympathetic activation is triggered to maintain systemic perfusion and oxygen delivery to vital organs. Adverse effects of catecholamine overactivation in sepsis include tachycardia-induced myocardial damage [2], inflammatory cytokine production [3], insulin resistance [4], and thrombogenicity [5]. Of note, tachycardia occurring with sepsis can increase the cardiac workload and result in myocardial oxygen consumption.

The use of β-adrenergic blockade is beneficial in patients with diverse cardiovascular diseases. In the recent decades, it has emerged as a possible treatment option in early sepsis to blunt the overwhelming adrenergic responses of cardiogenic [2, 6], metabolic [7], immunological [8], and coagulopathic [5] derangement. In animal models, β-blocker administration during sepsis appears to reduce the heart rate (HR) and adrenergic activation [9]. In a prospective study, esmolol use permitted the maintenance of target HRs within the range of 80–94 bpm, increased stroke volumes, and improved 28-day survival in septic patients [10]. An observational study revealed that patients with sepsis who had been prescribed β-blockers before admission had significantly lesser mortality [11]. Other clinical studies also suggest that premorbid β-blocker exposure has beneficial effects on sepsis outcomes [12, 13]. However, data on the effects of different types of β-blocker (*β1-selective* and non-selective) on sepsis outcomes are scarce. This study was conducted to investigate the impacts of premorbid *β1-selective* and non-selective β-blocker use on sepsis outcomes using data from a single medical center. We hypothesized that mortality after sepsis development would be lesser among patients who used β-blockers, especially *β1-selective* β-blockers, in the premorbid period.

## Materials and methods

### Patient selection and data collection

This retrospective single-center study was conducted with data from patients admitted to the medical or surgical ICU of the Taipei Veterans General Hospital, a tertiary medical center, between December 2015 and July 2017. Selected subjects’ medical records, including all accessible records of hospitalization, outpatient visits, prescriptions, and examinations, were reviewed. The following data were collected: (1) age, sex, and comorbidities; (2) source of infection and severity of sepsis; and (3) laboratory measurements obtained at the time of ICU admission. The arterial blood gas samples were used for determination of pH, PaO_2_, PaCO_2_, and HCO_3_^−^. PaO_2_/FiO_2_ ratio (PF ratio) was calculated as PaO_2_ divided by FiO_2_ at the time PaO_2_ was measured. Acute Physiology and Chronic Health Evaluation II (APACHE II) scores were calculated within 24 hours after ICU admission to evaluate disease severity [14]. Glasgow Coma Scale (GCS) score was recorded by the ICU physicians upon patients admitted to our ICU. The lowest mean arterial blood pressure (BP) and highest HR within 24 h after ICU admission were recorded. The study protocol is in accordance with the Helsinki Declaration and international ethical standards and was approved by the hospital’s ethics board (Num. 2017-09-018BC).

We included consecutive patients aged ≥ 18 years who were admitted to the ICU with the diagnosis of sepsis and fulfilled the Sepsis-3 criteria [1]. We considered patients who had been prescribed β-blockers for >1 month before ICU admission to be premorbid β-blocker users. We classified β-blockers as β1-selective (bisoprolol, metoprolol, atenolol) and non-selective (carvedilol, propranolol, labetalol, and acebutolol) [15].

### Outcome measurement

The primary outcome was to evaluate the association between previous β-blocker prescription and all-cause mortality in the ICU. Secondary outcomes were the amount of fluid resuscitation and norepinephrine usage (defined as any dose of norepinephrine administration to keep mean BP>65 mmHg) in the first 24 h of ICU admission, lactate concentrations at 0 and 6 h after ICU admission, duration of ventilator use, and ICU stay duration. All patients were followed for 28 days or until death.

### Statistical analysis

We express continuous variables as medians ± standard deviations. Student’s *t* test and analysis of variance were used to compare continuous variables. We express categorical values as absolute numbers with percentages; statistical comparisons were made using the *chi*-*squared test.* Cox proportional-hazards regression analysis was performed to investigate independent associations between clinical variables and ICU mortality. Variables with significant associations in the univariable analysis were adjusted for in a final multivariable regression model. To investigate the effects of premorbid *β-*blocker use modified by different conditions, we performed subgroup analyses with the cohort stratified by comorbidities and septic shock [1]. The survival curve was plotted using the Kaplan-Meier method with the statistical significance examined by the log-rank test. Two-tailed *P* values < 0.05 were considered to be significant. The data were analyzed using IBM SPSS Statistics 23 (SPSS Inc., Chicago, IL, USA) and MedCalc 19.1 (MedCalc Software, Mariakerke, Belgium).

## Results

### Study population and baseline characteristics

**Fig. 1:**
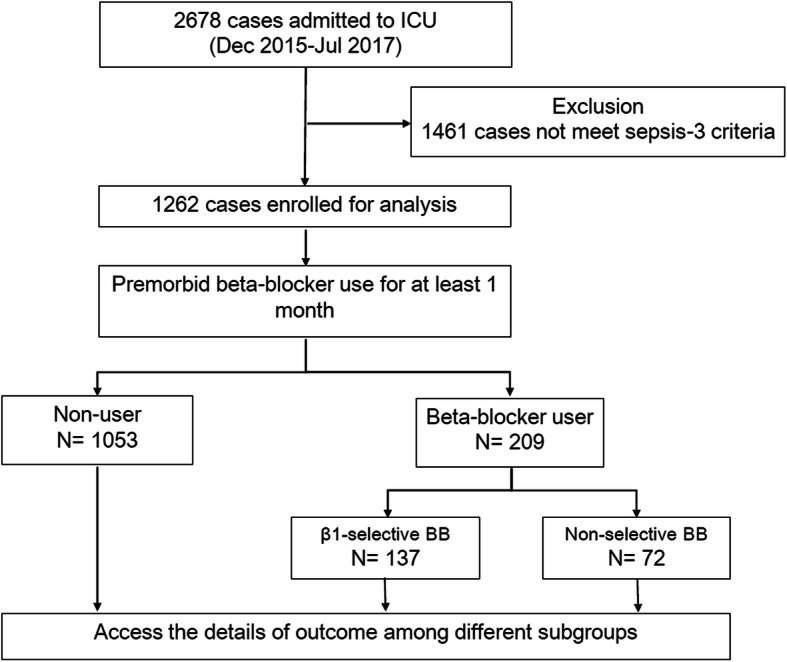
Flowchart of patient enrollment and classification

*Of 2678 cases assessed, 1262 subjects fulfilled the Sepsis-3 criteria. In total, 209 (16.6%) patients were premorbid* β-blocker users and 1053 patients had no previous β-blocker exposure. Of the 209 users, 137 patients took *β1-selective* and 72 patients took non-selective β-blockers. Figure 1 is the flowchart of patient enrollment and classification. Patient characteristics according to β-blocker use are presented in Table 1. Hypertension, diabetes mellitus, end-stage renal disease (ESRD), cirrhosis, heart failure, arrhythmia, and coronary artery disease were more prevalent among subjects with premorbid β-blocker exposure. Hypertension and coronary artery disease were more prevalent, and liver cirrhosis was less prevalent, among *β1-selective* than among non-selective β-blocker users. During initial ICU admission, patients with premorbid exposure to *β1-selective* β-blockers had lower HRs than did those with no exposure. Disease severity, reflected by APACHE II scores, did not differ among the three groups. There was also no significant difference of hemogram, including white blood cell count (WBC), hemoglobin, platelet count, serum electrolytes, arterial blood gas, and PF ratio, between the three groups (Table 1). The missing data of each variables were reported in the Supplement Table 1.

**Table 1:** Baseline characteristic of septic patients grouped by the status of premorbid beta-blocker usage

Variables	Nonusers *n*= 1053	Nonselective BB *n*= 72	β1-selective BB *n*= 137	***P*** value
Age, y/o	68.89±17.30§	66.71±16.73§	73.70±12.88†‡	0.003
Male (*n*, %)	700(66.5%)	45(62.5%)	94(68.6%)	0.673
APACHEII Score	26.53±8.46	26.86±7.72	26.45±7.33	0.939
Underlying disease (*n*, %)				
Hypertension	474(45.0%)‡§	43(59.7%)†§	101(73.7%)†‡	<0.001
DM	345(32.8%)‡§	35(48.6%)†	63(46.0%)†	<0.001
ESRD	86(8.2%)‡§	17(23.6%)†	19(13.9%)†	<0.001
Cirrhosis	85(8.1%)‡	12(16.7%)†§	7(5.1%)‡	0.014
Heart failure	98(9.3%)‡§	13(18.1%)†	26(19.0%)†	<0.001
Arrhythmia	100(9.5%)§	11(15.3%)	21(15.3%)†	0.043
CAD	112(10.6%)§	13(18.1%)§	53(38.7%)†‡	<0.001
COPD	74(7.0%)	4(5.6%)	9(6.6%)	0.881
Cancer, solid tumor	308(29.2%)	16(22.2%)	35(25.5%)	0.321
Cancer, hematologic	89(8.5%)§	3(4.2%)	4(2.9%)†	0.038
Autoimmune disease	58(5.5%)	8(11.1%)	10(7.3%)	0.124
Infectious source (*n*, %)				
Pneumonia	628(59.6%)	46(63.9%)	91(66.4%)	0.262
UTI	141(13.4%)	11(15.3%)	23(16.8%)	0.522
Blood stream infection	163(15.5%)	11(15.3%)	15(10.9%)	0.375
IAI	285(27.1%)	18(25.0%)	28(20.4%)	0.245
Soft tissue infection	88(8.4%)	6(8.3%)	10(7.3%)	0.914
Vital signs & lab data				
HR, beats/min	116.40±23.16§	114.89±25.91	109.52±22.74†	0.005
Mean BP, mmHg	61.31±13.58‡	67.72±19.22†§	62.64±11.99‡	0.001
GCS score	8.89±4.17	9.60±4.07	9.23±3.78	0.276
WBC count, 10^3^/μL	12.8±14.0	11.6±10.4	11.6±6.6	0.507
Hemoglobin level, g/dL	9.40±2.10	8.95±1.95	9.55±2.05	0.153
Platelet count, 10^3^/μL	158.3±114.4	132.2±88.9	166.5±78.6	0.111
Na, mmol/L	139.7±7.89	138.6±6.33	139.2±6.33	0.387
K, mmol/L	3.95±0.92	4.09±0.91	4.03±0.81	0.365
C-reactive protein	13.15±10.67	14.27±11.52	12.90±9.20	0.657
Albumin, mg/dl	2.83±0.79	2.92±0.45	2.94±0.55	0.209
pH	7.43±0.09	7.44±0.08	7.42±0.08	0.553
pCO_2_, mmHg	33.67±12.14	34.14±10.18	32.32±9.87	0.416
HCO_3_, mmol/L	21.22±5.30	21.76±4.30	20.60±4.55	0.272
PF ratio	270.7±149.1	243.1±134.9	271.9±136.2	0.399

^†^Significant difference (*P*<0.05) compared to the nonusers group

^‡^Significant difference (*P*<0.05) compared to the nonselective BB group

^§^Significant difference (*P*<0.05) compared to the β1-selective BB group

*BB β-blocker*, *APACHE* Acute Physiology and Chronic Health Evaluation, *DM* diabetes mellitus, *ESRD* end stage renal disease, *CAD* coronary artery disease, *COPD* chronic obstruction pulmonary disease, *UTI* urinary tract infection, *IAI* intra-abdominal infection, *GCS* Glasgow Coma Scale, *HR* heart rate, *BP* blood pressure, *WBC* white blood cell, *PF* ratio PaO_2_/FiO_2_ ratio

### Premorbid β-blocker use and clinical outcomes

**Fig. 2:**
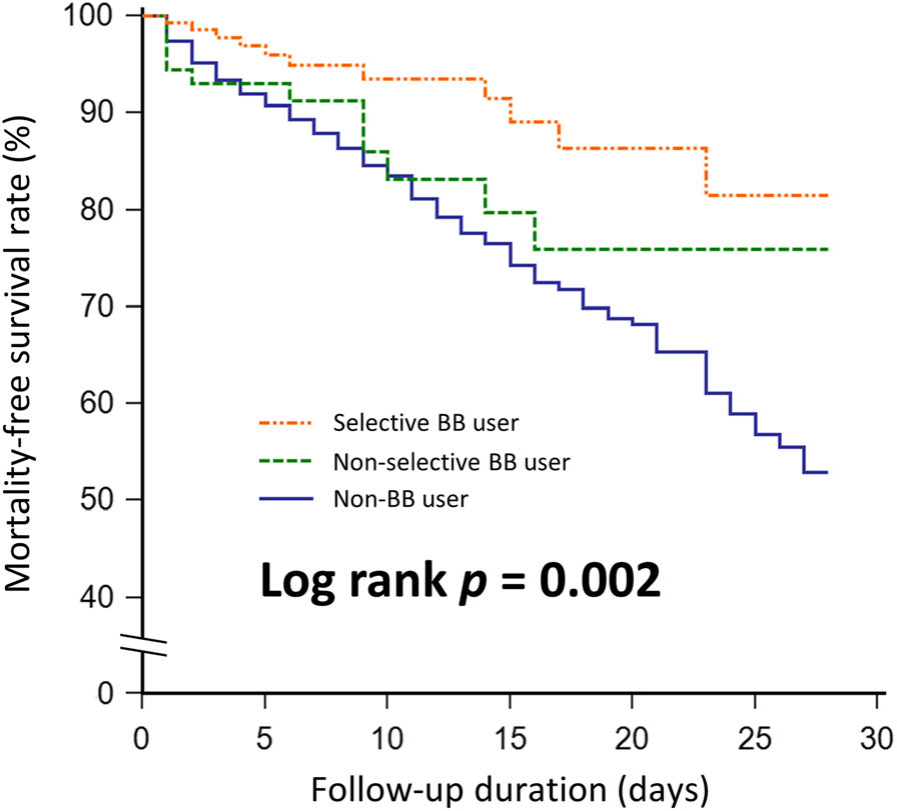
Kaplan–Meier curves of freedom from 30-day mortality in patients grouped by the status of premorbid beta-blocker (BB) usage

Compared with non-users, premorbid *β1-selective* β-blocker users had significant lower ICU mortality. Premorbid *β1-selective* β-blocker use also contributed to lower percentage of norepinephrine usage and lower lactate concentrations at 0 and 6 h after ICU admission. The total amount of fluid infusion, ICU stay, and days of ventilator use did not differ among the three groups (Table 2). In univariate Cox regression analysis, reduced 28-day mortality was associated with *β1-selective* [hazard ratio, 0.36; 95% confidence interval (CI), 0.19–0.68; P = 0.002; Table 3], but not non-selective β-blocker use. Higher HRs and lower arterial mean BP also were associated with greater ICU mortality. In the multivariate regression analysis adjusted for age, APACHE II score, hypertension, diabetes, hematological malignancy, HR, mean BP, and white blood cell count, *β1-selective* β-blocker exposure remained associated independently with lesser ICU mortality (*adjusted hazard ratio, 0.40; 95% CI, 0.18–0.92; P = 0.030*). A Kaplan–Meier curve also showed that premorbid *β1-selective* β-blocker exposure was associated with better 28-day survival (log-rank P = 0.002; Fig. 2).

**Table 2:** Outcomes of septic patients grouped by the status of premorbid beta-blocker usage

Variables	Nonusers *n*= 1053	Nonselective BB *n*= 72	β1-selective BB *n*= 137	***P*** value
Fluid infusion, L/24h	5.32±5.57	4.33±4.08	4.33±4.74	0.055
Norepinephrine use	485(46.1%)§	25(34.7%)	47(34.4%)†	0.008
Lactate, 0 h, mg/dL	25.25±27.67§	18.84±19.85	18.04±15.32†	0.004
Lactate, 6 h, mg/dL	24.18±26.40§	19.59±20.18	16.13±12.16†	0.001
Ventilator use, days	13.60±34.23	13.13±13.29	14.44±19.74	0.956
Long term ventilator use (*n*, %)	61(5.8%)	4(5.6%)	8(5.8%)	0.996
Length of ICU stay, days	9.82±6.90	11.29±8.51	10.81±7.73	0.087
ICU mortality (*n*, %)	217(20.6%)§	11(15.3%)	13(9.5%)†	0.005

^†^Significant difference (*P*<0.05) compared to the nonusers group

^‡^Significant difference (*P*<0.05) compared to the nonselective BB group

^§^Significant difference (*P*<0.05) compared to the β1-selective BB group

*BB β-blocker*, *ICU* intensive care unit

**Table 3:** Multivariate Cox regression analysis for the usage of beta blockers and incidence of mortality in the intensive care unit

	Univariate		Multivariate*	
	Crude HR	***P*** value	Adjusted HR	***P*** value
β1-selective BB usage	0.36(0.19–0.68)	0.002	0.40(0.18–0.92)	0.030
Nonselective BB usage	0.75(0.39–1.44)	0.384		
Age	0.99(0.98–0.99)	0.024	1.00(0.99–1.01)	0.972
Male	0.98(0.72–1.33)	0.887		
APACHEII	1.12(1.10–1.15)	<0.001	1.04(1.01–1.07)	0.014
Hypertension	0.66(0.49–0.88)	0.005	0.88(0.59–1.33)	0.550
DM	0.68(0.50–0.93)	0.016	0.72(0.48–1.10)	0.130
ESRD	1.00(0.62–1.63)	0.989		
Cirrhosis	1.50(0.93–2.41)	0.095		
CHF	1.20(0.77–1.86)	0.429		
Arrythmia	0.85(0.52–1.39)	0.512		
CAD	0.75(0.48–1.17)	0.206		
COPD	0.87(0.48–1.56)	0.634		
Cancer, solid tumor	1.04(0.76–1.43)	0.817		
Cancer, hematologic	4.12(2.68–6.36)	<0.001	2.47(1.38–4.42)	0.002
Autoimmune disease	1.23(0.70–2.18)	0.474		
Pneumonia	0.97(0.74–1.28)	0.848		
UTI	0.79(0.51–1.21)	0.273		
Blood stream infection	1.26(0.90–1.77)	0.174		
IAI	1.13(0.84–1.52)	0.413		
Soft tissue infection	1.04(0.75–1.44)	0.808		
HR	1.02(1.02–1.03)	<0.001	1.02(1.01–1.02)	<0.001
Mean BP	0.96(0.94–0.97)	<0.001	0.98(0.96–0.99)	0.007
GCS score	0.85(0.82–0.88)	0.851	0.92(0.88–0.97)	0.003
WBC count	1.00(1.00–1.00)	0.041	1.00(1.00–1.00)	0.338
Hemoglobin level	0.82(0.76–0.88)	<0.001	0.87(0.79–0.96)	0.005
Platelet count	1.00(1.00–1.00)	<0.001	1.00(1.00–1.00)	0.003
Na	0.99(0.97–1.01)	0.356		
K	1.13(0.97–1.33)	0.113		
C-reactive protein	1.01(0.99–1.02)	0.446		
Albumin	0.81(0.63–1.05)	0.110		
pH	0.02(0.01–0.10)	<0.001	0.81(0.10–6.76)	0.844
pCO_2_	1.01(0.99–1.02)	0.477		
HCO_3_	0.94(0.91–0.97)	<0.001	0.96(0.93–1.01)	0.064
PF ratio	1.00(1.00–1.00)	<0.001	1.00(1.00–1.00)	0.002

*Adjusted for variables with *P* < 0.05 in the univariate analysis

*HR* hazard ratio, *APACHE* acute physiology and chronic health evaluation, *DM* diabetes mellitus, *ESRD* end stage renal disease, *CHF* chronic heart failure, *CAD* coronary artery disease, *COPD* chronic obstruction pulmonary disease, *UTI* urinary tract infection, *IAI* intra-abdominal infection, *GCS* Glasgow Coma Scale, *HR* heart rate, *BP* blood pressure, *WBC* white blood cells, *PF ratio* PaO_2_/FiO_2_ ratio, *BB* beta blocker

### Subgroup findings

The results of subgroup analyses are shown in Table 4. Compared with non-use, premorbid β1-selective β-blocker use was associated with lesser ICU mortality, regardless of the presence or absence of hypertension, diabetes, ESRD, cirrhosis, heart failure, arrhythmia, coronary artery disease, cancer, and septic shock. No significant interaction between any of these variables and β1-selective β-blocker use was detected.

**Table 4:** Subgroup analysis of the relationship between premorbid beta blocker usage and mortality in the intensive care unit

	β1-selective BB vs. non-usage Crude OR	***P*** effect	***P*** interaction
Hypertension			
No	0.46(0.16–1.32)	0.150	0.725
Yes	0.36(0.16–0.81)	0.013	
DM			
No	0.46(0.22–0.98)	0.043	0.408
Yes	0.25(0.08–0.83)	0.024	
ESRD			
No	0.44(0.23–0.84)	0.012	0.998
Yes		0.998	
Cirrhosis			
No	0.35(0.18–0.69)	0.002	0.712
Yes	0.54(0.06–4.77)	0.581	
COPD			
No	0.31(0.15–0.61)	0.001	0.073
Yes	1.64(0.30–8.93)	0.569	
CHF			
No	0.38(0.19–0.76)	0.006	0.706
Yes	0.27(0.06–1.24)	0.092	
Arrythmia			
No	0.35(0.17–0.69)	0.003	0.702
Yes	0.48(0.10–2.25)	0.351	
CAD			
No	0.31(0.13–0.73)	0.007	0.475
Yes	0.51(0.18–1.45)	0.206	
Cancer, solid tumor			
No	0.40(0.20–0.82)	0.012	0.546
Yes	0.25(0.06–1.05)	0.058	
Cancer, hematologic			
No	0.44(0.23–0.84)	0.013	0.999
Yes		0.990	
Septic shock			
No	0.33(0.12–0.92)	0.034	0.576
Yes	0.48(0.21–1.11)	0.086	

*BB β-blocker*, *OR* odds ratio, *DM* diabetes mellitus. *ESRD* end-stage renal disease, *CHF* chronic heart failure, *CAD* coronary artery disease, *COPD* chronic obstruction pulmonary disease

## Discussion

In this retrospective study of data from 1262 septic patients, ICU mortality was lower among patients with premorbid β1-selective β-blocker exposure. Compared with non-use, premorbid β1-selective use was associated with lower lactate concentrations and lower percentage of norepinephrine use. Only β1-selective β-blocker use was associated with an improvement in 28-day ICU mortality. This study is the first to illustrate the effects of premorbid exposure to different types of β-blocker on short-term mortality among septic patients. The findings encourage long-term β1-selective β-blocker use, but prospective studies are needed to confirm the protective effect of such use in septic patients.

Tachycardia increases the cardiac workload and myocardial oxygen consumption. The shortening of the diastolic filling time during tachycardia decreases the stroke volume and coronary perfusion, contributing to the reduction of the ischemic threshold. Elevated HRs are associated with increased mortality in critically ill patients [16, 17], as shown in this study, and a survival benefit of *β*_*1*_*-adrenergic selective blockade* has been found in animal models [9]. By decreasing the HR, *β*-blockers decrease myocardial oxygen consumption and prolong the diastolic time and coronary perfusion, reducing the risk of myocardial ischemia. Several studies have shown that diastolic dysfunction is present in about half of septic patients and is a significant predictor of mortality [18]. Β-blockers have been shown to improve the diastolic function of patients with heart failure [19].

Nevertheless, the treatment of tachycardia during septic shock remains controversial. In the early phase of septic shock, tachycardia compensates for any reduction in cardiac output; HR reduction may interfere with this physiological response, reducing cardiac output and improving oxygen delivery [20]. However, tachycardia that persists after adequate resuscitation may represent sympathetic overstimulation. In patients with tachycardia (HR > 95 bpm) who received a titrated esmolol infusion with the goal of reducing the HR to 80–94 bpm, decreased HRs were offset by increased ventricular filling time and volume, ultimately resulting in increased stroke volume, which compensated for the HR decrease [10]. Similar hemodynamic effects of *β*_*1*_-adrenergic selective blockade by esmolol administration have been reported [21, 22]. With adequate preloading, HR reduction improves cardiac performance and efficiency [23], with the maintenance or even increase of the stroke volume. In our study, long-term β1-selective β-blocker users had significantly lower baseline HRs on ICU admission than did non-selective β-blocker users; this difference may translate into better outcomes.

Mechanisms other than HR reduction may explain the better sepsis outcomes associated with β-blocker use. The physiological response to stress includes the increased release of catecholamine. The early phase of sepsis is typically characterized by high cardiac output with decreased vascular tone, tachycardia, and impaired myocardial function. All of these factors can be associated with the elevation of the adrenergic drive to increase global and microvascular blood flow and oxygen delivery to vital organs. The direct cardiotoxic effects of catecholamine, especially norepinephrine, had been recognized for decades. A sustained increase in cardiac adrenergic drive adversely affected myocardial biology and structure phenotype in a heart failure model. The treatment of cardiac myocytes with norepinephrine caused a 60% loss of these cells [24], and the exposure of cardiac myocytes to isoproterenol had similar effects [25]. Several animal studies have demonstrated the occurrence of β1-adrenergic receptor signaling, which is considered to be more harmful to cardiac myocytes than is β2-adrenergic receptor signaling [25, 26]; these findings suggest that β_1_-adrenergic receptor signaling is the key mechanism for adrenergic-driven cardiotoxicity. In a clinical trial, differences in β_1_-adrenergic and β_2_-adrenergic receptor blocking doses indicated that β_*1*_adrenergic selective blockade had a better treatment effect for heart failure [27]. Previous studies have shown that activation of Na/K ATPase, which is stimulated by catecholamine, enhances glycolytic turnover and increases lactate production [28, 29]. Our findings were consistent with these results that premorbid β-blocker use had lower lactate production, probably due to the reduction of β-stimulation; and we found that only β1-selective rather than nonselective β-blocker had this effect. Hence, chronic β-blocker use may contribute to systemic protection from the catecholamine surge that occurs during sepsis.

*H*yperproduction of NO by the inducible form of NO synthase (iNOS) may contribute to the hypotension and vascular hyporeactivity during septic shock [30]*.* Downregulation of alpha1-receptor expression also contributed to hypotension in the septic animal models [31, 32]. Esmolol infusion decreased the iNOS expression in vascular tissues [32, 33], and up-regulated mRNA expression of alpha1-receptors [32] in experimental septic shock models. In our study, we found a lower norepinephrine requirement in the β1-selective β-blocker group, which could be due to the improvement of vascular function caused by the β1-selective β-blocker. The lower vasopressor requirement also protected patients from potential side effects of high-dose catecholamine. The improved vascular function may translate to better tissue perfusion, and the lower lactate levels in the β1-selective β-blocker group.

Esmolol also improves coagulation and microvascular circulation, as determined by assessment of the sublingual microcirculatory blood flow [21]. During sepsis, physiological anticoagulation and fibrinolytic mechanisms are impaired, and the coagulation pathway shifts toward a pro-coagulant state [5]. Coagulation system dysregulation causes the dissemination of intravascular coagulation, leading to microcirculatory dysfunction and tissue production at the cellular level [17]. β_1_- and β_2_-adrenergic receptors act differently on coagulation functions. β_2_-adrenergic stimulation suppresses platelet aggregation [34]. β_1_-adrenergic stimulation inhibits fibrinolysis by reducing prostacyclin synthesis [35], whereas β_2_-adrenergic stimulation promotes tissue plasminogen activator release, leading to enhanced fibrinolytic activity. Thus, β1-selective β-blocker may reduce platelet activation via relative β_2_-adrenergic activation, and enhance fibrinolysis through increased plasminogen activation and prostacyclin synthesis [36]. In the present study, premorbid β-blocker users had lower baseline lactate levels than did non-users. After initial resuscitation, more premorbid β1-selective than non-selective β-blocker users achieved >10% lactate clearance, suggesting that β1-selective β-blockers could possibly play a role in enhancing microcirculation function by improving the pro-coagulation state during sepsis.

β_1_- and β_2_-adrenergic receptors also seem to have different actions on the immune system. Th1 cells stimulate macrophages and natural killer T cells, and the production of pro-inflammatory cytokines, whereas Th2 cells have the opposite actions, inhibiting macrophage activation and T cell proliferation. Th1, but not Th2, cells have β_2_-adrenergic receptors. Hence, β_2_-receptor stimulation suppresses Th1 cell activation with a relative increase in the Th2 cell response [2]. Thus, selective β_1_-blockade could promote β_2_-adrenergic pathway activation and contribute to the suppression of the pro-inflammatory status. In septic animal models, esmolol reduced the levels of the pro-inflammatory cytokine tumor necrosis factor (TNF)-α in blood [6] and peritoneal fluid [37]. Metoprolol reduced the hepatic expression of proinflammatory cytokines and the plasma interleukin (IL)-6 level [9]. In contrast, the non-selective β-blocker propranolol enhanced inflammation and increased the TNF-α and IL-6 levels [38, 39]. The serum levels of anti-inflammatory cytokines, such as IL-10, are increased with stimulation by the selective β_1_-blocker atenolol [8] and by β_2_-blockers [40]. Hence, the benefits of β-blockers may also be immune mediated. Selective β-blockers have anti-inflammatory effects, which could explain the better sepsis outcomes in chronic β1-selective β-blocker users in this study.

Postmorbid usage of β-blockers after sepsis established was reported to improve circulatory and metabolic status and reduce mortality [10, 23]. In most clinical trials, β-blockers were started after 24 h of ICU admission [10, 21, 22]. On the other hand, premorbid β-blocker usage before sepsis development was reported to provide survival advantage in database study [11] or experimental study [9]. Ackland et al. found better protective effect of β-blocker, with reduction of proinflammatory cytokines, once it was given before septic insult than after induction of endotoxemia [9]. Our study provided clinical evidence for the benefit of premorbid β-blocker use in septic patients. We postulated that long-term, premorbid β-blocker use may increase patients’ tolerance to the excessive catecholamine surge during acute stress and contribute to hemodynamic or metabolic benefits long before sepsis occurred. Further prospective studies are needed to delineate the optimal timing of initiating β-blocker therapy.

Our findings are in line with previous findings that premorbid β-blocker exposure is associated with the improvement of outcomes in patients with sepsis [11–13]. Contrary to our findings, Singer et al. [12] reported that the mortality rate was lower among patients with premorbid exposure to non-selective β-blockers than among those with premorbid β1-selective β-blocker exposure. However, their study was based on Medicare administrative data, with patient inclusion in 2009–2011 according to ICD-9 diagnostic codes for sepsis, septic shock, and systemic inflammatory response syndrome, without consideration of clinical markers such as laboratory values and vital signs. In the present study, we used the Sepsis-3 criteria for patient inclusion, and considered a broad range of clinical information and data dating to 2015–2017, when sepsis management was more in line with treatment guidelines.

This study has several limitations. First, as it was retrospective, we could not determine the causal relationship between premorbid β1-selective β-blocker exposure and mortality. Second, it was based on the review of medical records from a single center. Disease severity was greater in our sample than in previous samples; thus, the observed benefits of β1-selective β-blockers in terms of sepsis outcomes may not extend to all septic patients. Third, the types of β-blocker prescribed were distributed unevenly; β1-selective β-blockers are preferred in our region when β-blocker use is indicated, and non-selective β-blocker use is predominant for certain diseases, such as liver cirrhosis, which may have caused bias. We attempted to correct for such bias by adjusting the multivariate regression and subgroup analyses for comorbidities. Fourth, as previous mentioned, β-blocker can influence the platelet and coagulation functions. However, we do not routinely evaluate platelet function or coagulation factors in the daily practice. Troponin-I, which is a useful marker to indicate myocardial injury, was also not routinely measured. We did not adjust it in the analysis since there was too much missing data of coagulation factors and troponin-I. Finally, we only collected the data from the point of ICU admission, which may had been treated partially in the emergency department or ordinary ward.

## Conclusions

Our findings suggest that premorbid *β1-selective*, but not non-selective, β-blocker use is associated with lower ICU mortality among septic patients. The protective effect of *β1-selective* β-blockers may be related to their role in the suppression of the overwhelming adrenergic response, enhancement of cardiac performance, improvement of vascular and microcirculation dysfunction, and anti-inflammatory effects. The results of this study increase our knowledge of the β-adrenergic activity during sepsis. Prospective studies are needed to confirm the therapeutic potential of *β1-selective* β-blocker use in septic patients.

## Supplementary Information

**Additional file 1: Supplemental Table.** Numbers of study subjects with missing data.

## Data Availability

The datasets generated and analyzed are available from the corresponding author on reasonable request.

## Abbreviations

ICU: Intensive care unit
*PF ratio*: *PaO*_*2*_*/FiO*_*2*_ *ratio*
APACHE II: Acute Physiology and Chronic Health Evaluation II
*GCS*: *Glasgow Coma Scale*
BP: Blood pressure
*WBC*: *White blood cell*
CI: Confidence interval
ESRD: End-stage renal disease
HR: Heart rate
ICU: Intensive care unit
IL: Interleukin
TNF: Tumor necrosis factor
