# Supplementary material for: Premorbid β1-selective (but not non-selective) β-blocker exposure reduces intensive care unit mortality among septic patients
Source: J Intensive Care. 2021 May 13;9:40. doi: 10.1186/s40560-021-00553-9 (PMC8116825; doi:10.1186/s40560-021-00553-9)
Supplement: Supplementary file 1 — Additional file 1: Supplemental Table. Numbers of study subjects with missing data. [file 40560_2021_553_MOESM1_ESM.docx]

**Supplemental Table.** Numbers of study subjects with missing data

| Variables | **Subjects with missing data**  N=1262 |
| --- | --- |
| Age | 0 |
| Male | 0 |
| APACHEII Score | 1 |
| Underlying disease |  |
| Hypertension | 0 |
| DM | 0 |
| ESRD | 0 |
| Cirrhosis | 0 |
| Heart failure | 0 |
| Arrhythmia | 0 |
| CAD | 0 |
| COPD | 0 |
| Cancer, solid tumor | 0 |
| Cancer, hematologic | 0 |
| Autoimmune disease | 0 |
| Infectious source |  |
| Pneumonia | 0 |
| UTI | 0 |
| Blood stream infection | 0 |
| IAI | 0 |
| Soft tissue infection | 0 |
| Vital signs & lab data |  |
| HR | 0 |
| Mean BP | 41 |
| GCS score | 0 |
| WBC count | 73 |
| Hemoglobin level | 57 |
| Platelet count | 71 |
| Na | 39 |
| K | 37 |
| C-reactive protein | 57 |
| Albumin | 69 |
| pH | 29 |
| pCO2 | 22 |
| HCO3 | 31 |
| PF ratio | 177 |
| Outcome |  |
| Fluid infusion | 0 |
| Norepinephrine use | 0 |
| Lactate, 0 h | 101 |
| Lactate, 6 h | 65 |
| Ventilator use | 0 |
| Long term ventilator use | 0 |
| Length of ICU stay | 0 |
| ICU mortality | 0 |

BB, β-blocker*;* APACHE, acute physiology and chronic health evaluation; DM, diabetes mellitus; ESRD, end stage renal disease; CAD, coronary artery disease; COPD, chronic obstruction pulmonary disease; UTI, urinary tract infection; IAI, intra-abdominal infection; GCS, Glasgow Coma Scale; HR, heart rate; BP, blood pressure; WBC, white blood cell; PF ratio, PaO2/FiO2 ratio *;* ICU, intensive care unit
